# Perinatal depression in migrant and refugee women on the Thai–Myanmar border: does social support matter?

**DOI:** 10.1098/rstb.2020.0030

**Published:** 2021-06-21

**Authors:** Gracia Fellmeth, Emma Plugge, Mina Fazel, Suphak Nosten, May May Oo, Mupawjay Pimanpanarak, Yuwapha Phichitpadungtham, Raymond Fitzpatrick, Rose McGready

**Affiliations:** ^1^ Nuffield Department of Population Health, University of Oxford, Richard Doll Building, Old Road Campus, Oxford OX3 7LT, UK; ^2^ Shoklo Malaria Research Unit, Mahidol Oxford Tropical Medicine Research Unit, 68/30 Bantung Road, PO Box 46, Mae Sot, Tak 63110, Thailand; ^3^ Health and Justice Team, Health Improvement Directorate, Public Health England, 60 Caversham Road, Reading RG1 7EB, UK; ^4^ Department of Psychiatry, University of Oxford, Warneford Hospital, Oxford OX3 7JX, UK; ^5^ Faculty of Tropical Medicine, Mahidol University, 420/6 Ratchawithi Road, Bangkok 10400, Thailand

**Keywords:** social support, depression, perinatal, migrant, refugee, low- and middle-income

## Abstract

Migrant and refugee women are at risk of perinatal depression due to stressors experienced before, during and after migration. This study assesses the associations between social support and perinatal depression among migrant and refugee women on the Thai–Myanmar border. We conducted a cohort study of pregnant and post-partum women. Depression status was assessed using a structured clinical interview. Received support, perceived support and partner support were measured in the third trimester. Logistic regression was used to calculate associations between social support measures and perinatal depression controlling for demographic, socio-economic, migration, obstetric and psychosocial factors. Four hundred and fifty-one women (233 migrants; 218 refugees) were included. The prevalence of perinatal depression was 38.6% in migrants and 47.3% in refugees. Migrants had higher levels of received, perceived and partner support than refugees. After controlling for all other variables, higher levels of received support remained significantly associated with a lower likelihood of perinatal depression in migrants (adjusted odds ratio 0.82; 95% CI 0.68–0.99). In both groups, depression history and trauma were strongly associated with perinatal depression. Our study highlights the importance of received social support to perinatal depression in migrant women on the Thailand–Myanmar border. The perinatal period offers a valuable opportunity to ask women about their support and offer community-level or public policy interventions to nurture support networks in current locations and resettlement destinations.

This article is part of the theme issue ‘Multidisciplinary perspectives on social support and maternal–child health’.

## 1. Introduction

Maternal mental disorders are a significant contributor to maternal morbidity worldwide with potentially severe consequences for women, their families and wider society [1,2]. The physical, psychological and societal adjustments that occur during pregnancy, labour and the transition to motherhood are extensive and can increase women's vulnerability to developing mental disorders [1,3]. Perinatal depression is the most common perinatal mental disorder, disproportionately affecting women living in poverty. Across low- and middle-income countries (LMIC), the estimated prevalence of perinatal depression is 19%, although estimates vary between settings [4].

Left untreated, perinatal depression is associated with distress and suffering and may limit women's ability to engage in paid work and provide care. At its most extreme, perinatal depression is associated with suicide—an important cause of maternal death across LMIC and high-income settings alike, particularly among marginalized populations [5–7]. Children of mothers with depression are more likely to experience a range of poor physical, cognitive and emotional outcomes [8]. In LMIC especially, these poor outcomes among children perpetuate the inter-generational cycle of social and economic deprivation.

Contributors to maternal mental disorders are complex, multifactorial and often context-specific. Risk factors across different settings include demographic factors such as age and ethnicity, socio-economic factors such as educational background and income, obstetric factors, psychosocial factors such as interpersonal violence (IPV) and wider, community-level factors such as access to and quality of healthcare [1,2]. One of the most consistent determinants of perinatal depression that has been highlighted across diverse settings is social support. Women who are pregnant or have recently given birth may experience an increased need for support to cope with the physical strains of pregnancy and labour, breastfeeding and caring for newborns and the range of emotional responses to motherhood [9]. A lack of support has been associated with poor maternal mental health, while strong and established support networks have been shown to buffer against stressors experienced by women [10].

Assessing social support is challenging. A complex and multi-dimensional concept, social support encompasses informational, instrumental, affiliative and emotional aspects [10–12]. Numerous measures of social support have been developed. Some focus on the objective: measures of received support, for example, assess the total practical, informational and emotional support available [11,13]). Other measures emphasize the subjective experience of support. Perceived support, for example, has been defined as the extent to which an individual perceives the support available to them to be sufficient to meet their needs [14]. While both received and perceived support have been associated with perinatal depression, evidence suggests that perceived sufficiency of support may be the greater predictor of mental state [14].

Perinatal women who are migrants or refugees are at risk of developing mental disorders as a result of the multiple psychological and socio-economic stressors endured before, during and after migration [15,16]. Access to healthcare—vital to ensuring a healthy pregnancy, safe delivery and postnatal support—is all too often lacking for migrant and refugee women who can remain marginalized in their destination settings [17]. Social isolation and the loss of family and social networks may be further exacerbated by language and cultural barriers in destination countries [18,19]. These multiple vulnerabilities create a complex cycle of challenges for migrant and refugee women and their families.

While migrant and refugee populations share many commonalities, there are also marked differences. Often, though not always, migrants exert more personal choice over the decision to move, relocating in hope of finding remunerative employment or in search of education or better futures for the children. For refugees, experiences of displacement are often more acute responses to immediate threats to life or livelihood. Levels of social support available may also differ significantly between migrants and refugees across different contexts. In order to better support displaced populations, a better understanding of the particular needs of each group is required. To date, research on migrant and refugee perinatal mental health has focused on high-income destination settings while women relocating from one LMIC to another are under-represented [18]. This paper addresses the evidence gaps by assessing the association between social support and perinatal depression among migrant and refugee women on the Thailand–Myanmar border.

### (a) Setting

Long-standing conflict in Myanmar between ethnic Burman-dominated central government and multiple ethnic minority groups has resulted in displacement of Myanmar populations to the border with Thailand. Displaced populations in this region include migrants and refugees. Migrants relocate primarily in search of employment and higher wages in agriculture, manufacturing and service industries on the Thai side of the border. Residing in villages and towns along both sides of the border, many of the estimated 500 000–1 million migrants make daily commutes across the Moei River which constitutes the border between Myanmar and Thailand [20]. Many migrants lack formal identification papers or work permits, rendering them prone to fines, exploitation or even arrest and deportation by the Thai authorities. Refugees, by contrast, live within established refugee camps on the Thai side of the border. There are currently an estimated 93 000 refugees living along the border, though in recent years, numbers have dwindled as a result of efforts to repatriate residents following Myanmar's quasi-return to democracy [21,22]. Refugees benefit from basic aid packages within the camps including shelter, food rations, education and healthcare provided by non-governmental organizations. However, movement into and out of the camps is severely restricted and many of the younger residents born within the camp have never left its perimeter. Work opportunities are limited and unlike migrants, most refugees have no means of earning an income.

This study was carried out at the Shoklo Malaria Research Unit (SMRU), a field station of the Mahidol Oxford Tropical Medicine Research Unit (MORU). Located in Tak Province, Thailand, SMRU has provided maternity services and conducted research along the Thailand–Myanmar border area since 1986. At the time of research, SMRU ran three clinics to the north and south of the border town of Mae Sot: Mawker Tai and Wang Pha clinics serve rural labour migrant families, while a clinic at Maela camp provided care to refugee women and infants.

## 2. Methods

A prospective cohort study exploring perinatal depression among labour migrant and refugee women on the Thai–Myanmar border was conducted between October 2015 and January 2018 [23]. Pregnant labour migrant and refugee women attending SMRU antenatal clinics at Maela, Wang Pha or Mawker Tai were invited to participate in the cohort study if they met the following inclusion criteria: they were aged 18 years or above; in their first trimester of pregnancy (defined as having an estimated gestational age less than 14 weeks on ultrasound dating scan); had a viable pregnancy; planned to deliver at SMRU; were willing and able to take part. Data collection occurred at four timepoints: first trimester (baseline), second trimester, third trimester and one month post-partum. Depression status was assessed at every visit; data were collected on demographic and socio-economic factors in the first trimester; on psychosocial and migration factors in the third trimester; and on obstetric factors at one month post-partum. Previous analyses exploring predictors of perinatal depression identified social support as a key determinant [24]. These previous analyses relied on a single, composite measure of social support. The current paper presents a secondary data analysis which explores the association in greater depth by including three measures of social support.

### (a) Social support

Social support was assessed in the third trimester of pregnancy. Three measures of social support were used: received social support, perceived sufficiency of support and partner support. *Received social support* was assessed using a modified version of Chen *et al*.'s [25] Social Support in the Postpartum Period Scale, which assesses the informational, emotional and practical support available to women. This scale was selected because of its breadth of scope, relevance to the local setting and previous use among a similar group of post-partum migrant women in Taiwan [25]. The scale was adapted to maximize its appropriateness and facilitate local administration. Wording was simplified and due to the challenges of Likert-type scales in our setting, the response scale was replaced with binary (yes/no) options. Four items from the original scale were removed because concepts proved either difficult to translate or differentiate from other items or because they were considered inappropriate for the local setting. The final modified scale included eight items with possible scores ranging from 0 to 8 and higher scores indicating higher levels of received support (electronic supplementary material, table S1). *Perceived sufficiency of support* was assessed through a single, direct question: ‘Do you feel you have enough support?’ and coded as a binary variable, with ‘yes’ indicating sufficient support and ‘no’ indicating insufficient support. *Partner support* was considered important following previous qualitative research in this setting, which identified a lack of partner support as a key aspect of women's narratives around mental wellbeing [26]. Partner support was assessed through a single, direct question: ‘Does your husband support, care for and understand you?’ and coded as a binary variable, with ‘yes’ indicating the presence of partner support and ‘no’ indicating the lack thereof.

### (b) Depression status

Depression was assessed using the Structured Clinical Interview for the Diagnosis of DSM-IV Disorders (SCID), a semi-structured interview based upon DSM-IV diagnostic criteria for depression [27,28]. The SCID provides a clinical diagnosis of depression and is thus distinct from screening instruments which are limited to identifying *symptoms* indicative of depression. The decision to use the SCID was based on prior research on the psychometric validity and acceptability of various screening tools in this setting [29,30]. The Likert-type response scales used by screening tools were unfamiliar and challenging to participants. Participants and local staff reported a preference for the SCID, which relies on open questions and provides opportunities for elaboration. Perinatal depression included depression of any severity and was defined as meeting the DSM-IV criteria for Major or Minor Depressive Disorder or Depressive Disorder ‘Not Otherwise Specified’ [28] at least once during the study period (i.e. in the first, second or third trimester of pregnancy or at one month post-partum). Interviews were conducted in Burmese, Sgaw Karen and Poe Karen by midwives employed by SMRU. The midwives are themselves members of the local migrant and refugee communities: besides having extensive clinical and research experience in this setting, they bring first-hand familiarity with the local cultural context, enabling high levels of trust with study participants. Depression diagnoses were independently reviewed by a clinician (G.F.) who was blinded to midwives’ diagnoses and discrepancies were discussed with a study psychiatrist (M.F.).

### (c) Demographic, socio-economic, migration, psychosocial and obstetric data

Data were collected on demographic, socio-economic, migration, obstetric and psychosocial factors which have been identified as risk factors for perinatal depression among migrant and refugee populations [20,31]. Variables and their categorization are shown in electronic supplementary material, table S2. A history of depression was self-reported by participants and assessed using a single, direct question: ‘Have you ever experienced depression in the past?’. Trauma history was assessed using an adapted version of the Trauma History Screen, with scores ranging from 0 to 9 and higher scores representing a greater number of traumatic events experienced [32]. The presence of IPV was assessed using the following question which forms part of standardized screening for domestic violence and abuse in the USA: ‘Does a partner, or anyone at home, hurt, hit or threaten you?’ [33]. The item was given a binary score, with ‘yes’ representing the presence of any form of IPV and ‘no’ representing no IPV.

### (d) Statistical analyses

The current analysis is limited to participants of the cohort study who attended the third trimester follow-up, when social support was assessed. Characteristics of participants included in this subgroup and those excluded from it were compared. Characteristics of participants included in the current analysis were summarized using medians and ranges for non-normally distributed continuous data and frequencies and percentages for binary and categorical data. Differences between migrant and refugee women were assessed using the Mann–Whitney *U* and χ^2^ tests. Social support measures were summarized using the median scores and interquartile ranges (received support) and the proportion of women responding ‘yes’ (perceived support and partner support). Pairwise correlations between social support measures were assessed with correlation coefficients less than 0.3 considered weak, 0.3–0.7 considered moderate and greater than or equal to 0.7 considered strong. Associations between social support and perinatal depression were assessed using logistic regression with separate models for migrants and refugees. First, univariable logistic regression was used to quantify the strength of association between each measure of social support individually and perinatal depression, providing unadjusted odds ratios (OR) with corresponding 95% confidence intervals (CI). Next, a series of multivariable logistic regression models were built using stepwise estimation. Pairwise correlation between exposure variables was assessed: for any pair of variables significantly (*p* < 0.05) and strongly (correlation coefficient ≥ 0.7) correlated, only the variable with the greater magnitude of association with perinatal depression in the univariable analysis was retained for inclusion in the multivariable model. Base models were limited to the three measures of social support only. Demographic, socio-economic, migration, obstetric and psychosocial variables were sequentially added in subsequent steps of the model and trends in the resulting OR compared. The extent to which each adjusted model improved upon the previous model was assessed using likelihood ratio (LR) *χ*^2^ tests and the absolute percentage change, along with Cox and Snell's and Nagelkerke's pseudo-*R*^2^. Analyses were carried out using STATA 14IC [34].

## 3. Results

### (a) Characteristics of study participants

The original cohort included 568 (318 migrants; 250 refugees) participants. Due to the mobile nature of this population, attendance at each follow-up varied. The current analysis is limited to the 451 participants (233 migrants; 218 refugees) who attended the third trimester follow-up and completed the social support assessment. A comparison of subgroup and full cohort participants is presented in electronic supplementary material, table S3. Electronic supplementary material, table S4 summarizes characteristics of migrant and refugee women included in the current analysis. The median age of participants was 25 years, half (50.6%) were Sgaw Karen and almost half (46.5%) had completed fewer than 3 years of education. Migrant women were more likely than refugees to be ethnic Burman (47.6 versus 0.5%), Buddhist (94.9 versus 40.8%) and in paid employment (81.9 versus 47.2%). Migrants had a shorter duration of living in their current location (median 5 versus 10 years), and more planned a return to Myanmar in the future (84.5% versus 2.8%). Fewer migrants had experienced previous depression (8.6 versus 47.3%) or traumatic events (37.3 versus 58.7%), but reports of IPV (7.8 versus 3.7%) were higher among migrants.

The prevalence of perinatal depression and levels of social support are presented in electronic supplementary material, table S5. The prevalence of perinatal depression was 38.6% (90/233) among migrants and 47.3% (103/218) among refugees. Migrants had a median received support score of 8; 88.8% (207/233) reported sufficient support and 86.3% (201/233) reported support from partners. Refugees had a median received support score of 4.5; 49.1% (107/218) reported sufficient support and 53.2% (116/218) reported support from partners. Electronic supplementary material, figure S1 shows the proportion of women who endorsed each item on the received support scale. Refugee women were less likely to endorse each item, with the greatest discrepancies seen in help with cooking, information on caring for their baby and having someone to talk to when they felt sad. Pairwise correlation between the three measures of support were positive and ranged in magnitude from 0.43 to 0.49 for the whole sample, 0.29 to 0.39 for migrants and 0.33 to 0.34 for refugees (electronic supplementary material, table S6).

### (b) Associations between social support and perinatal depression

Electronic supplementary material, table S7 shows the univariable associations between individual social support measures and perinatal depression. Among migrants, received and perceived support were statistically significantly associated with perinatal depression: the unadjusted odds ratio for perinatal depression associated with received support was 0.79 per unit increase in score (95% CI 0.68–0.92), while insufficient perceived support was associated with a 2.40 (95% CI 1.05–5.49) increase in the odds of perinatal depression. Not having a supportive partner was associated with a 1.48 (95% CI 0.70–3.14) increase in the odds of perinatal depression, though this association did not reach statistical significance. Among refugees, only perceived support was significantly associated with perinatal depression: insufficient perceived support was associated with a 1.89 (95% CI 1.10–3.24) increase in the odds of perinatal depression. The associations between received support (unadjusted OR 0.99 per unit increase in score; 95% CI 0.87–1.13) and partner support (unadjusted OR 1.23; 95% CI 0.72–2.10) with perinatal depression among refugees were not statistically significant.

Electronic supplementary material, table S8 presents our full model selection process and adjusted odds ratios for the multivariable regression analyses. Two pairs of variables were significantly inter-correlated among migrants and refugees: ethnicity with language, and parity with gravida. Of these, ethnicity and parity were retained for the multivariable analysis. In the migrant model, religion was strongly correlated with ethnicity and excluded from multivariable analysis. Reason for migrating was excluded due to the high number of missing values for this variable. Table 1 summarizes results of the multivariable regression analyses for the base (only social support measures) and final (full) models. Among migrants, when all three measures of social support were simultaneously entered into the model, only received support remained statistically significantly associated with perinatal depression (adjusted OR 0.79; 95% CI 0.68–0.92). In the final model for migrants, received support remained significantly associated with perinatal depression after controlling for all other variables in the model (adjusted OR 0.82; 95% CI 0.68–0.99; standardized OR for received support 0.64; 95% CI 0.42–0.97 as shown in electronic supplementary material, table S8). Along with received support, a history of depression (adjusted OR 6.70; 95% CI 1.38–32.74) and trauma score (adjusted OR 2.02; 95% CI 1.36–3.01) remained significantly associated with perinatal depression in migrants. Among refugees, in the base model, only perceived support remained significantly associated with perinatal depression (adjusted OR 1.89; 95% CI 1.10–3.24). In the final model for refugees, all three social support measures lost statistical significance and were dropped. The three variables which remained significant after controlling for all other variables in the final model for refugees were a history of depression (adjusted OR 3.35; 95% CI 1.78–6.33), trauma score (adjusted OR 1.63; 95% 1.23–2.17) and telephone ownership (adjusted OR 0.42; 95% CI 0.21–0.85).

**Table 1.** Odds ratios of associations between perinatal depression and social support measures: base and final models for migrant and refugee women.

model	variables	OR (95% CI)	*p*-value	Cox and Snell pseudo-*R*^2^	Nagelkerke's pseudo-*R*^2^
*migrant women*					
base	received support	0.79 (0.68–0.92)	<0.01	0.042	0.057
	perceived support	*dropped*	—		
	partner support	*dropped*	—		
final	received support	0.82 (0.68–0.99)	0.04	0.160	0.216
	perceived support	*dropped*	—		
	partner support	*dropped*	—		
	history of depression	6.70 (1.38–32.74)	0.02		
	trauma score	2.02 (1.36–3.01)	0.01		
*refugee women*					
base	received support	*dropped*	—	0.027	0.036
	perceived support	1.89 (1.10–3.24)	0.02		
	partner support	*dropped*	—		
final	received support	*dropped*	—	0.179	0.239
	perceived support	*dropped*	—		
	partner support	*dropped*	—		
	history of depression	3.35 (1.78–6.33)	<0.01		
	trauma score	1.63 (1.23–2.17)	<0.01		
	telephone ownership	0.42 (0.21–0.85)	0.02		

## 4. Discussion

The burden of perinatal depression of any severity was high in our population, affecting over one in three migrants (38.6%) and almost one in two refugees (47.3%). Our definition of perinatal depression includes mild depressive episodes, and comparisons with prevalence estimates from other studies—many of which focus exclusively on severe depression—must be made with caution. Nevertheless, the finding that such a significant proportion of women experience depression of some severity during the perinatal period is alarming.

Levels of social support were high among migrants, of whom the majority reported having sufficient support (89%) and supportive partners (86%). The median received support score for migrants was 8—the maximum possible score. By contrast, only half of refugee women reported having sufficient support (49%) or supportive partners (53%). At first, these findings may seem surprising: given the precarious nature of migrants’ daily lives on the one hand, and the relative security and material aid available to refugee women on the other. However, there may be deeper factors underlying our findings. For migrants in this setting, a move to the border area is often seen as temporary, with the specific purpose of seeking employment and higher wages. Migration provides a means to securing a better future for their families and—to some extent—migrants exert a degree of choice over where to live and when to return to Myanmar. Many migrants move as a family unit and as such may benefit from practical and emotional support provided by relatives. Others leave children under the care of grandparents in Myanmar. Among these families, despite the lower levels of practical support received, women experience a form of social support from their own parents looking after their children. There may be a strong sense of family solidarity conferred by husband and wife moving to find work and financially support children and grandparents. It is perhaps such a feeling of family ‘togetherness’ that is reflected in the high levels of support reported by migrants in our study. By contrast, refugees’ decision to leave Myanmar—which is often rendered more acute by instances of ethnicity-based discrimination and violence—is coupled with a wish to resettle permanently in ‘third’ countries such as Australia, the UK and the USA. However, resettlement is not guaranteed and with the option of returning to Myanmar deemed unsafe, many refugees find themselves living in uncertainty. Compared with migrants, refugees may feel they have less agency over their lives. Many refugee families are geographically dispersed with members either left behind in Myanmar or resettled abroad. This uncertainty and family fragmentation may result in a fragility that leaves refugee women feeling unsupported, particularly during pregnancy and the post-partum period when the need for family support is greatest.

Received, perceived and partner support showed moderate positive correlation to one another, suggesting that although the three measures are aligned there are likely to be additional influencing factors. The correlation between perceived and received support, for example, suggests that other factors beyond the receipt of support influence the perception of support. Similarly, the correlation between partner support and perceived and received support suggests that wider support networks beyond the marital partner are important to women.

Examining the unadjusted associations between individual measures of social support and depression showed that lower levels of received and perceived support were associated with a higher likelihood of perinatal depression among migrants, and perceived support was associated with a higher likelihood of perinatal depression among refugees. The association between partner support and perinatal depression failed to reach statistical significance in either group. When we explored the associations of the three measures of support simultaneously, only received support remained significantly correlated with depression in migrants: each unit increase in received support scores was associated with a 20% reduction in the odds of perinatal depression. Among refugees, only perceived support remained correlated with depression: perceived insufficiency of support was associated with a 90% increase in the odds of perinatal depression. Comparisons between received and perceived support and their respective associations with perinatal depression have been subject to much research, with perceived support the more consistently and strongly associated with mental health [11,13,35]. Our findings suggest that the importance of each measure may vary across populations. We can only speculate on possible reasons underlying our finding. One possible explanation is that among migrants, absolute levels of practical support—whether in the form of childcare or help with household work—have a direct impact upon women's availability to engage in paid labour, as it frees them of domestic and caring responsibilities. The notion of *sufficiency* of support in this group may be less relevant in the context of practical help being so essential to securing a daily wage. For refugee women, on the other hand, practical support with childcare or housework perhaps plays a less important role, as there is little opportunity for employment and less urgency to earn an income. Among refugees, therefore, received support may be less closely associated with mental state, while the role of perceived sufficiency of support gains in significance. More research is required to better understand the mechanisms underlying these differences between migrant and refugee groups.

Controlling the association between social support and perinatal depression for additional variables highlighted further interesting correlations. Among migrants, received support remained significantly associated with perinatal depression after controlling for demographic, socio-economic, migration, obstetric and psychosocial factors in the final model: each increase in received support score was associated with a 20% reduction in perinatal depression (adjusted OR 0.82). Alongside received support, previous depression (adjusted OR 6.70) and trauma score (adjusted OR 2.02) were significantly associated with perinatal depression in the final model for migrants; both of these variables had large effect sizes compared with received support (adjusted standardized OR 0.64). Interestingly, for refugees, no measures of social support remained significant in the final adjusted model. In refugees, the factors remaining in the final model were previous depression (adjusted OR 3.35), trauma score (adjusted OR 1.63) and telephone ownership (adjusted OR 0.42).

Previous depression is a well-established risk factor for perinatal depression, and in itself, the association we found is unsurprising [1,2]. Of note, however, is the identification of this association using a single-item self-report measure of prior depression. The strength of association, along with the persistence of this variable in the full model after controlling for all other variables across both migrant and refugee models, suggests that asking women directly about their mental health history may offer a reliable measure in our context and may help identify women at risk of perinatal depression. The finding that experience of trauma is also associated with a greater likelihood of perinatal depression among migrants and refugees mirrors findings from other vulnerable populations across LMIC settings [1,18]. We used telephone ownership as a proxy measure for disposable income. Our findings suggest that owning a telephone was associated with a halving of the odds of depression. Among refugee women in particular, telephones also offer an important means of staying connected with family members. Owning a telephone may represent a lifeline that has greater significance among fragmented refugee families than among migrants. When the association between social support and perinatal depression was controlled for demographic, socio-economic and migration factors only (steps 2–4 of the multivariable model), being Muslim emerged as a factor associated with depression among refugee women. Muslim women constitute a minority group within Maela refugee camp and are often subject to prejudice from other camp residents, most of whom are Christian or Buddhist. We are not aware of any published research on the particular vulnerabilities of Muslim women within refugee camps in this setting; the vulnerability of Muslim refugee women to perinatal depression represents an important research priority.

Our findings highlight important commonalities and differences in factors associated with perinatal depression among migrant and refugee women in our setting. Migrant and refugee women attending maternity care should be asked about levels of social support available to them. Other studies have highlighted the importance of assessing both the degree of social support available to perinatal women and the perceived sufficiency of this support [36]. In our setting, it may be appropriate to focus efforts on eliciting levels of received support among migrants. Both migrant and refugee women should be systematically and sensitively screened for previous depressive disorders and trauma, with a view to conducting more in-depth risk assessments for perinatal depression in those who have a history of either. Early identification is key, and regular follow-up of and support for women identified as being at high risk of perinatal depression is necessary to prevent its onset and minimize its impact. As Spry *et al*. discuss in their piece [37], interventions promoting social support antenatally can reduce rates of depression postnatally. Although in our setting, partner support appeared to play a less significant role than perceived and received support, other studies have found that increasing partners’ involvement in women's care can enhance support to women [38].

Local support groups may provide a means of offering meaningful help to at-risk women [37]. Perinatal migrant and refugee women should be encouraged to build up support networks. Peer support groups have been shown to be helpful for the prevention and management of perinatal depression in LMIC, with the added benefits of bringing women together and widening mechanisms of support [39]. McLeish & Redshaw [40], for example, found that emotional, informational and practical support from trained volunteers benefited perinatal women from disadvantaged backgrounds—including those with insecure immigration status—in the UK, helping to reduce social isolation and building women's confidence, self-esteem and self-efficacy [40]. Initiatives delivered by trained members of the local community may be particularly useful if women are reluctant to seek care from health professionals, or when access to health services is limited [36,41]. Importantly, the need for support during the perinatal period changes over time, and determining what forms of support are truly helpful to women would facilitate enhanced care for some of the most vulnerable mothers [9].

### (a) Strengths and limitations

This is the first study to assess multiple forms of social support among populations living on the Thailand–Myanmar border. Our participants are a marginalized and geographically mobile group of women including many with informal status in Thailand who are often excluded from research. By including both migrant and refugee women from the same setting, our findings allow for direct comparison between these two groups. The three measures of social support allow for different aspects of this multi-dimensional construct to be explored simultaneously. The use of a diagnostic interview rather than a screening tool for the ascertainment of depression represents a further strength, providing clinical diagnoses of depression rather than relying on symptoms of the disorder.

There were a number of limitations to our study. Our definition of depression applied in this analysis includes depression of any severity. Although we consider this a strength, it means that comparisons with other studies—many of which focus exclusively on severe depression—must be made with caution. A history of depression was assessed through self-report and may have been subject to bias. Formal diagnoses of mental disorders are rare in this setting where mental health services are scarce, and we had no alternative means of establishing depression histories. However, the fact that depression history remained correlated with depression in the final model for both migrants and refugees gives confidence in the utility of this measure. The use of single-item measures for perceived and partner support may have limited response variability. The prevalence of IPV was low and we may have underestimated the true burden due to the lack of detailed assessment. Perinatal depression was a composite measure of depression experienced any time during the perinatal period. The exposure measured did not precede the outcome and our data, therefore, cannot discern causality of the association between social support and perinatal depression, nor the directionality of the association. We cannot determine whether low levels of social support might lead to depression because women find it difficult to cope without support, or whether depression might be negatively influencing women's perceptions of support.

## 5. Conclusion

Our findings highlight the importance of received social support to the perinatal mental health of migrant women on the Thailand–Myanmar border. For both migrant and refugee women, a history of depression and trauma experience were strongly associated with perinatal depression. Migrant and refugee women face unique challenges and many may find it difficult to develop support networks in resettlement destinations. Culturally sensitive antenatal and postnatal services which encourage and facilitate help-seeking are needed to better support women from marginalized communities. Given that pregnancy is for many women a time of increased contact with health services, the perinatal period offers a valuable opportunity to assess women's support networks and provide interventions that can assist in helping these women better develop and nurture social networks.

## Data accessibility

The dataset generated for this study is not publicly available due to the sensitive nature of the information and the vulnerable legal status of the participants. Data can, therefore, only be accessed by submission of a request to the Data Access Committee at Mahidol Oxford Tropical Medicine Research Unit (MORU), Bangkok, Thailand. The data sharing policy is available at http://www.tropmedres.ac/data-sharing. Send requests to Dr Phaik Yeong Cheah (phaikyeong@tropmedres.ac).
